# Role of interpersonal tactile and visual interaction in modulating postural sway and enhancing stability

**DOI:** 10.1038/s41598-026-49999-7

**Published:** 2026-05-14

**Authors:** Daiju Ikawa, Akito Miura, Kohei Miyata, Kazutoshi Kudo

**Affiliations:** 1 Department of Occupational Therapy, School of Rehabilitation, Tokyo Professional University of Health Sciences, 2-22-10, Shiohama, Koto, 135-0043 Tokyo Japan; 2 Faculty of Human Sciences, Waseda University, Tokorozawa, Saitama, Japan; 3 Center for Brain Science, RIKEN, Wako, Saitama, Japan; 4 Department of Life Sciences, Graduate School of Arts and Sciences, The University of Tokyo, 3-8-1, Komaba, Meguro, 153-8902 Tokyo Japan

**Keywords:** Detrended fluctuation analysis, Interpersonal touch, Postural stability, Recurrence quantification analysis, Sensory information, Health care, Neuroscience

## Abstract

Manual support and interpersonal interaction can enhance postural stability during upright standing, yet it remains unclear how tactile and visual interactions influence the temporal organization of postural control. We used a 2 × 2 factorial design manipulating interpersonal touch (Touch vs. No Touch) and partner visibility (Visible vs. Invisible) while participants stood quietly in pairs. Center-of-pressure velocity time series were analyzed using linear and nonlinear methods, including recurrence quantification analysis (RQA) and detrended fluctuation analysis (DFA). Both tactile interaction and partner visibility were associated with reduced sway magnitude, with no significant interaction between modalities. Nonlinear analyses revealed that tactile interaction was associated with reduced recurrence and lower DFA scaling exponents across timescales, indicating altered temporal organization. Visual coupling influenced recurrence measures and longer-timescale structure but did not significantly affect short-timescale scaling. These findings suggest that tactile and visual interactions contribute additively to sway reduction, yet are associated with distinct patterns of temporal organization in postural control.

## Introduction

The human standing posture is a motor behavior that plays a significant role in many daily activities; however, it is inherently unstable and often requires support in developmental and rehabilitative contexts. In such contexts, manual support plays a critical role in stabilizing the upright posture. For example, parents assist toddlers as they take their first steps, rehabilitation therapists support patients during therapy, and caregivers help prevent falls in older adults. These interventions facilitate the acquisition, recovery, and maintenance of postural stability^1,2^. The stabilizing effects of manual support arise from both physical contact and sensory input, particularly interpersonal light touch^3–7^. Even minimal fingertip contact with an external reference reduces center-of-pressure (CoP) variability, suggesting that tactile information can enhance balance in the absence of substantial mechanical support^8–10^. Interpersonal light touch further extends this effect to bidirectional contexts, where both individuals exhibit reduced postural sway during light contact^3–7^.

Postural stabilization can also be achieved in the absence of direct physical contact. Visual information about body orientation relative to the environment reduces postural sway^11^, and face-to-face visual coupling between individuals has been shown to synchronize and stabilize postural fluctuations^12–14^. These findings collectively demonstrate that both tactile and visual information are associated with enhanced stability during interpersonal interaction.

While these findings demonstrate both enhanced postural stability and interpersonal coordination, it remains unclear how tactile and visual interactions reorganize the intrinsic temporal structure of individual postural fluctuations. Because tactile and visual information differ fundamentally in their temporal and spatial characteristics, they may influence postural control through distinct mechanisms. Tactile information provides rapid, localized feedback about the relative position and movement of individuals, operating at shorter timescales and with higher spatial precision^5^. In contrast, visual information provides broader contextual cues about the partner’s overall postural state but is characterized by longer neural processing delays and lower spatial resolution^14^. These modality-specific properties may produce differences in postural control dynamics that are not fully captured by conventional linear (i.e., static) measures of postural sway, such as the standard deviation (SD) or root mean square (RMS) of the CoP time series.

To examine these differences, we employed two complementary nonlinear (i.e., dynamic) analytical techniques: recurrence quantification analysis (RQA) and detrended fluctuation analysis (DFA)^15,16^. RQA characterizes the recurrence and stability of temporal patterns in a time series by quantifying the extent to which similar states reappear over time^16,17^. DFA quantifies the scaling properties of temporal structures, revealing long-range temporal correlations and self-similar structures across multiple timescales^18,19^. Together, these methods allow us to determine whether interpersonal tactile and visual interactions merely reduce sway magnitude or uniquely reorganize the temporal structure of individual postural sway.

**Fig. 1:**
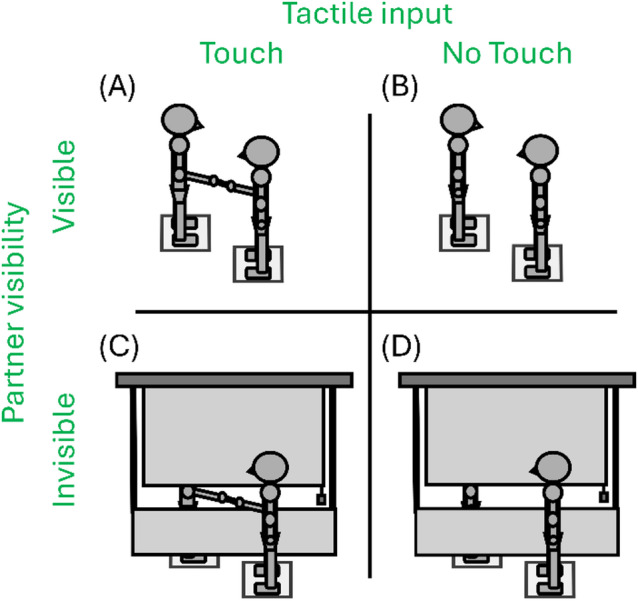
Overview of the four experimental conditions: (**A**) Visible Touch (VT), (**B**) Visible No Touch (VNT), (**C**) Invisible Touch (IT), and (**D**) Invisible No Touch (INT).

The present study used a 2 × 2 factorial design manipulating interpersonal tactile input (Touch vs. No Touch) and partner visibility (Visible vs. Invisible) (Fig. 1). We hypothesized that both modalities would reduce sway magnitude but would differentially modulate the temporal and scale-dependent properties of postural control due to their distinct spatiotemporal processing characteristics. This design allowed us to determine whether tactile and visual cues exert additive or distinct effects on postural control dynamics.

## Results

Twenty-four healthy adults (12 sex-matched pairs) participated in the study. Using a 2 × 2 factorial design, we manipulated partner visibility (visible vs. invisible) and tactile input (touch vs. no touch), resulting in four conditions: Visible Touch (VT), Visible No Touch (VNT), Invisible Touch (IT), and Invisible No Touch (INT). Participants stood quietly on separate force platforms while anteroposterior (AP) CoP velocity time series were recorded, and all subsequent analyses were performed using these AP sway data. The primary measures were the root mean square (RMS), recurrence quantification analysis (RQA; %RECUR, LMEAN), and detrended fluctuation analysis (DFA; short- and long-term α). Detailed procedures for each analysis are described in the Methods section.

### RMS

**Fig. 2:**
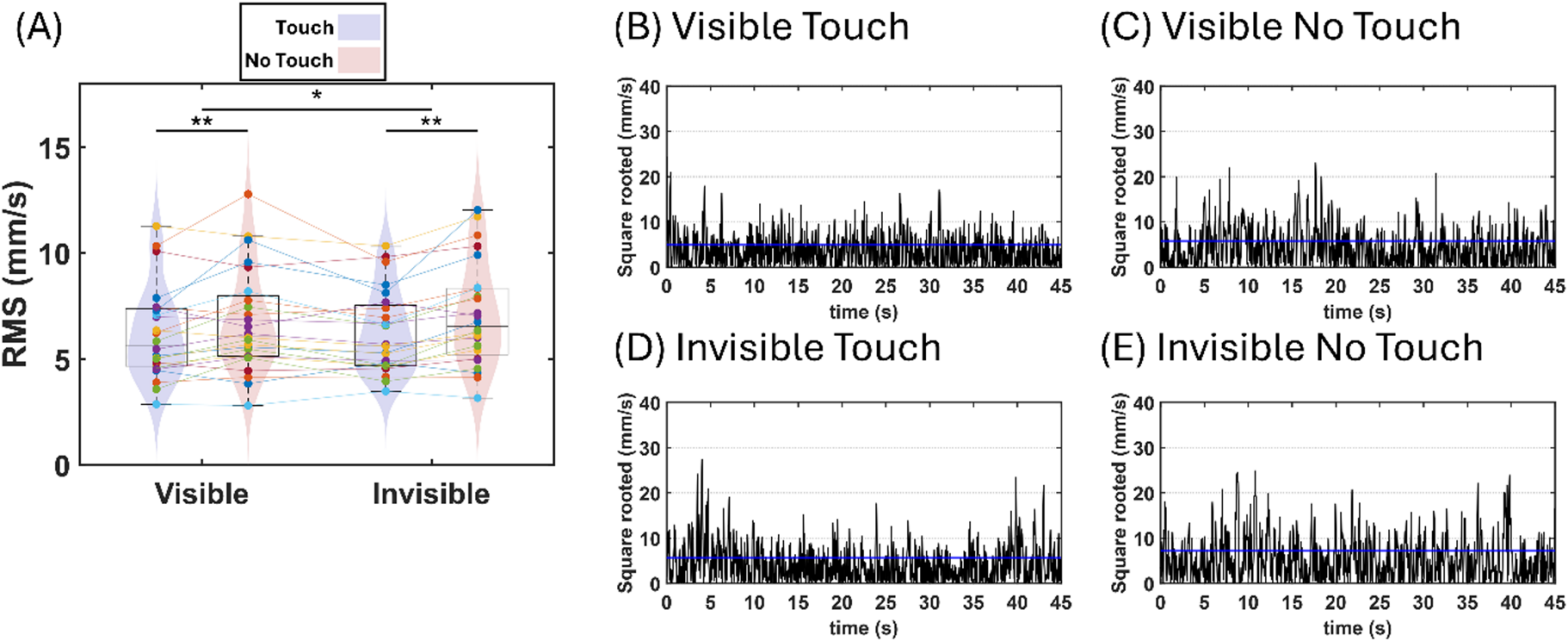
RMS results across experimental conditions: (**A**) Violin plots represent the data distribution for each condition, with the width indicating the probability density. Box plots overlay the violin plots, showing the median and interquartile range. Each data point, represented as a colored dot, corresponds to an individual participant, with a unique color assigned to each pair. Panels (**B**) to (**E**) Representative examples from the same participant under each condition: (**B**) Visible Touch, (**C**) Visible No Touch, (**D**) Invisible Touch, and (**E**) Invisible No Touch.

The RMS results indicated that both tactile input and partner visibility contributed to reducing the variability of the CoP velocity time series (Fig. 2A). A two-way repeated-measures ANOVA revealed a significant main effect of tactile input (*F*(1, 23) = 14.18, *p* = 0.001, ηp² = 0.3814). This result confirms that tactile input significantly decreased RMS compared to the no-touch condition. Additionally, a significant main effect of partner visibility was observed (*F*(1, 23) = 4.49, *p* = 0.045, ηp² = 0.1635), showing that RMS was significantly lower when the partner was visible than when they were invisible. No significant interaction between tactile input and partner visibility was found (*F*(1, 23) = 2.79, *p* = 0.108, ηp² = 0.1083). While both modalities significantly reduced sway variability, the effect size for tactile input was larger than that for partner visibility.

### RQA

**Fig. 3:**
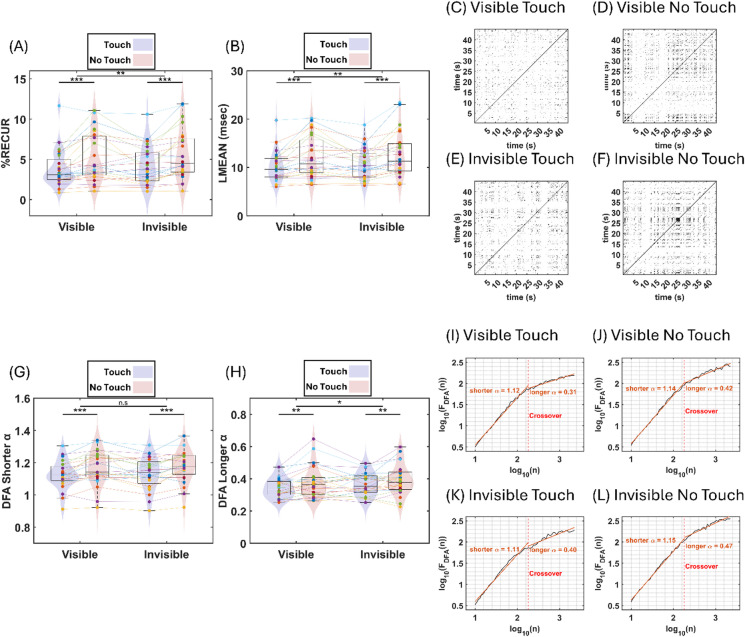
Violin plots show RQA and DFA results across conditions: (**A**) %RECUR and (**B**) LMEAN for RQA, and (**G**) DFA Shorter $\:\alpha\:$ and (**H**) DFA Longer $\:\alpha\:$ for DFA. The width of the violin plots represents data distribution, with box plots indicating the median and interquartile range. Each data point represents an individual participant, color-coded by pair. Panels (**C**) to (**F**) show representative examples from the same participant under each condition for RQA (%RECUR and LMEAN): (**C**) Visible Touch, (**D**) Visible No Touch, (**E**) Invisible Touch, and (**F**) Invisible No Touch. Panels (**I**) to (**L**) show corresponding representative examples for DFA (DFA Shorter $\:\alpha\:$ and DFA Longer $\:\alpha\:$) from the same participant under the same conditions: (**I**) Visible Touch, (**J**) Visible No Touch, (**K**) Invisible Touch, and (**L**) Invisible No Touch.

The ANOVA revealed a significant main effects for both tactile input (*F*(1, 23) = 17.27, *p* = 0.0004, ηp² = 0.4289) and partner visibility (*F*(1, 23) = 9.68, *p* = 0.0049, ηp² = 0.2961) on %RECUR (Fig. 3A). Specifically, %RECUR was lower in the tactile input condition than in the no-touch condition, and similarly lower when the partner was visible compared to when they were invisible. No significant interaction was found (*F*(1, 23) = 0.00, *p* = 0.962, ηp² = 0.0001). While both modalities modulated sway dynamics, the effect size for tactile input was larger.

Similar patterns were observed for LMEAN (Fig. 3B), with significant main effects of tactile input (*F*(1, 23) = 16.66, *p* = 0.0005, ηp² = 0.4200) and partner visibility (*F*(1, 23) = 8.98, *p* = 0.0064, ηp² = 0.2808). There was no significant interaction (*F*(1, 23) = 0.09, *p* = 0.761, ηp² = 0.0041). Consistent with the %RECUR results, a larger effect size was observed for tactile input than for partner visibility.

### DFA

The DFA revealed a distinct crossover phenomenon characterized by different short- and long-term scaling behaviors (Fig. 3G and H). Specifically, shorter α exceeded 1 under all conditions, whereas longer α consistently remained below 0.5.

The ANOVA revealed significant main effect of tactile input (*F*(1, 23) = 15.47, *p* = 0.0007, ηp² = 0.4019) on the shorter $\:\alpha\:$ scaling exponent. Although the effect of partner visibility did not reach the threshold for statistical significance (*F*(1, 23) = 3.63, *p* = 0.0695, ηp² = 0.1356). No significant interaction was found (*F*(1, 23) = 0.07, *p* = 0.7938, ηp² = 0.0000). While tactile input significantly modulated these short-term dynamics, its effect size was larger than that for partner visibility.

Regarding the longer $\:\alpha\:$ scaling exponent, there were significant main effects for both tactile input (*F*(1, 23) = 8.50, *p* = 0.0078, ηp² = 0.2703) and partner visibility (*F*(1, 23) = 7.03, *p* = 0.014, ηp² = 0.2348). No significant interaction was observed (*F*(1, 23) = 0.21, *p* = 0.650, ηp² = 0.0076). Notably, in contrast to the results for the shorter $\:\alpha\:$, partner visibility also had a significant effect on the longer $\:\alpha\:$.

## Discussion

This study examined whether tactile and visual interactions exert additive or distinct influences on postural control dynamics during upright standing. Linear analysis using RMS confirmed that both tactile input and partner visibility reduced postural sway variability (Fig. 2A), consistent with previous findings^3–7,13^. The absence of a significant interaction between these factors, combined with the substantially larger effect size for tactile input, indicates that these two sensory sources provide additive rather than redundant contributions to postural stabilization through distinct control mechanisms.

Beyond linear measures, nonlinear analyses revealed qualitative differences in temporal organization. Tactile interaction was associated with significant reductions in %RECUR and LMEAN (Fig. 3A, B). Reduced recurrence does not necessarily imply a loss of postural stability; rather, lower recurrence has been associated with more flexible postural regulation in skilled populations such as dancers^20^, whereas higher recurrence rates has been linked to more rigid and stereotyped patterns of postural control^21^. Specifically, the decrease in LMEAN observed in the present study may indicate a reduction in the duration of repeated postural patterns, implying shorter sequences of similar postural adjustments^22^. This reduction in temporal persistence suggests a shift toward more transient and flexible postural regulation under tactile interaction rather than reliance on prolonged, stereotyped adjustment.

DFA analyses further clarified how these changes manifested across different timescales. Tactile interaction was associated with significantly lower DFA α values across short and long timescales (Fig. 3G and H). Such anti-persistence reflects more frequent corrective reversals and has been interpreted as consistent with velocity-based control^19^, although the functional meaning of DFA α appears context-dependent, with both higher and lower values reported in association with enhanced motor performance across different tasks and populations^15,16,23^. Thus, the present findings suggest that tactile interaction is associated with a reorganization of temporal control structure rather than a simple increase or decrease in stability.

A different pattern emerged for visual coupling. While partner visibility significantly reduced recurrence measures (i.e., %RECUR, LMEAN) and the longer $\:\alpha\:$ exponent (Fig. 3A, B, and H), it did not significantly affect the short-term scaling exponent, despite reducing overall sway magnitude. This pattern may reflect differences in temporal response characteristics between visual and tactile systems, with visual processing generally associated with slower response dynamics^24^. Together, these findings suggest that tactile and visual interactions are associated with distinct temporal patterns of postural regulation. While tactile interaction was linked to stronger short-timescale adjustments, visual coupling appeared more related to longer-timescale coordination.

Interpersonal light touch may also be understood as a suprapostural task that constrains whole-body sway^8,25^. Maintaining light contact with a partner requires stabilizing the limb relative to the point of contact, and such task-level constraints have been shown to reduce postural variability even when stability is not the primary goal of the task. From this perspective, reduced sway during interpersonal light touch may reflect task-dependent reorganization of postural control rather than the effect of tactile feedback alone^26,27^.

In addition, passive mechanical coupling arising from body compliance, such as elasticity or viscosity of the upper limbs, may contribute to the observed changes in sway dynamics. Studies of passive-dynamic systems and embodied control suggest that morphological and mechanical properties of the body can substantially shape movement dynamics independent of explicit neural regulation^28,29^. Because interaction forces and whole-body kinematics were not directly measured in the present study, the relative contributions of active sensory-motor processes and passive mechanical effects cannot be fully disentangled. Future research incorporating motion capture and direct measurements of contact forces will be necessary to clarify how task constraints, sensory processes, and mechanical coupling jointly contribute to interpersonal postural stabilization.

## Conclusions

These findings highlight that tactile and visual coupling offer complementary forms of sensory input for postural control. Tactile interaction was associated with immediate and localized adjustments, enhancing responsiveness to small perturbations, whereas visual input contributes to slower integrative control over extended timescales. Importantly, our results underscore that manual support while standing cannot be elucidated by examining physical contact alone; rather, stabilizing effects emerge through the interplay of multiple sensory modalities, each engaging a distinct dynamic mechanism. This dissociation between touch and vision provides novel insights into the sensory organization of postural control and may inform the development of rehabilitation protocols or assistive strategies that enhance balance through modality-specific sensory engagement. Future studies should systematically manipulate gaze direction and fixation targets, while also incorporating force measurements, to isolate and clarify the specific contributions of visual access and mechanical interactions in modulating postural stability.

## Methods

### Participants

Twenty-four healthy adults (18 men and 6 women; mean age = 24.5 years, SD = 2.8) participated in this study. The sample size was determined using a power analysis performed with G*Power 3.1. Based on a repeated-measures ANOVA, a total sample size of 24 was required to detect a medium effect size (Cohen’s f = 0.25) with a power (1 – $\:\beta\:$) of 0.80 and an $\:\alpha\:$ level of 0.05. Participants were paired according to the criterion that both individuals were of the same sex and were acquainted, forming a total of 12 pairs. The mean height difference between the paired participants was 3.9 cm (SD = 3.0). The Ethics Committee of the Graduate School of Arts and Sciences at the University of Tokyo approved this study (ethical approval numbers: 625 and 625-2). All methods were performed in accordance with the relevant guidelines and regulations, including the Declaration of Helsinki. Written informed consent was obtained from all participants prior to the experiment.

### Apparatus

Two separate force platforms (SPORTS SENSING Co., Ltd., Tokyo, Japan) were used to record the CoP position by measuring force and moment along the three axes. Analog signals were sampled at 1,000 Hz using an analog-to-digital converter (USB-6218 BNC, National Instruments, Inc., Austin, TX, USA) and synchronized using LabVIEW software (LabVIEW 2014, National Instruments, Inc., Austin, TX, USA) to ensure precise recording timing between the two platforms.

### Experimental design and procedure

The experiment used a 2 × 2 factorial design to investigate the effects of partner visibility and tactile input. Partner visibility was manipulated at two levels—visible and invisible—while tactile input was varied at two levels—interpersonal touch and no touch. These factors resulted in four experimental conditions: Visible Touch (VT), Visible No Touch (VNT), Invisible Touch (IT), and Invisible No Touch (INT) (Fig. 1).

For the invisible conditions, roll screens were suspended from the ceiling and secured with clips and cords to block the partner from view, ensuring a controlled environment. In the interpersonal touch condition, participants flexed their right elbows to approximately 90°, with their right shoulders slightly externally rotated and lightly touching their partner’s right index finger. In the No Touch condition, participants were instructed to stand with their arms at their sides. To assess the potential confounding effect of arm posture, we conducted a preliminary pilot test in which participants maintained the same raised arm position without physical contact. The results suggested that postural sway in this condition was comparable to that observed in the standard no-touch condition, indicating that differences in arm configuration alone are unlikely to account for the stabilizing effects observed during interpersonal touch. Based on this, the arms-at-side posture was adopted for all no-touch conditions. The force platforms were positioned diagonally, face-to-face, to accommodate each pair’s physical characteristics, ensuring that their right index fingers touched while maintaining consistent relative positioning across all conditions, with a mean diagonal distance of 118.67 cm (SD = 7.17) between the centers of their heels.

The experiment consisted of five sets, each consisting of one trial for each of the four conditions. The trial order within each set was randomized, ensuring that each condition was performed five times. Rest periods of approximately 5 min were provided between sets to minimize the potential influence of fatigue on postural sway.

The participants stood barefoot on separate force platforms, with their heels positioned 5 cm apart and feet parallel. Each trial lasted 60 s. In all conditions, participants were instructed to focus on a visual mark at eye level to maintain consistent visual fixation and avoid direct eye contact with their partner. Data analysis focused on the 45-second segment from 15 to 60 s, excluding the initial adjustment period, to capture the interval when interpersonal touch was expected to stabilize the participants’ CoP time series.

### Data analysis

All CoP data were analyzed using MATLAB (R2024a, MathWorks Inc., Natick, MA, USA). All CoP position time-series data were filtered using a fourth-order Butterworth low-pass filter with a 10 Hz cutoff frequency^4,5^ and then differentiated to obtain the CoP velocity time-series data. After differentiation, the data were decimated from 1,000 Hz to 100 Hz to facilitate computational efficiency without losing the relevant information^16,30^. The analysis focused on CoP velocity time-series data in the AP direction because balance is primarily regulated by adjustments in CoP velocity rather than CoP position^19^, and interpersonal touch effects are primarily observed in CoP velocity time-series in the AP direction when individuals are positioned facing each other with their right shoulders aligned^5^. The CoP velocity time series in the AP direction was analyzed to calculate RMS as a linear measure and RQA and DFA as nonlinear measures, with detailed procedures for each described in the following sections.

### Root Mean Square (RMS)

**Fig. 4:**
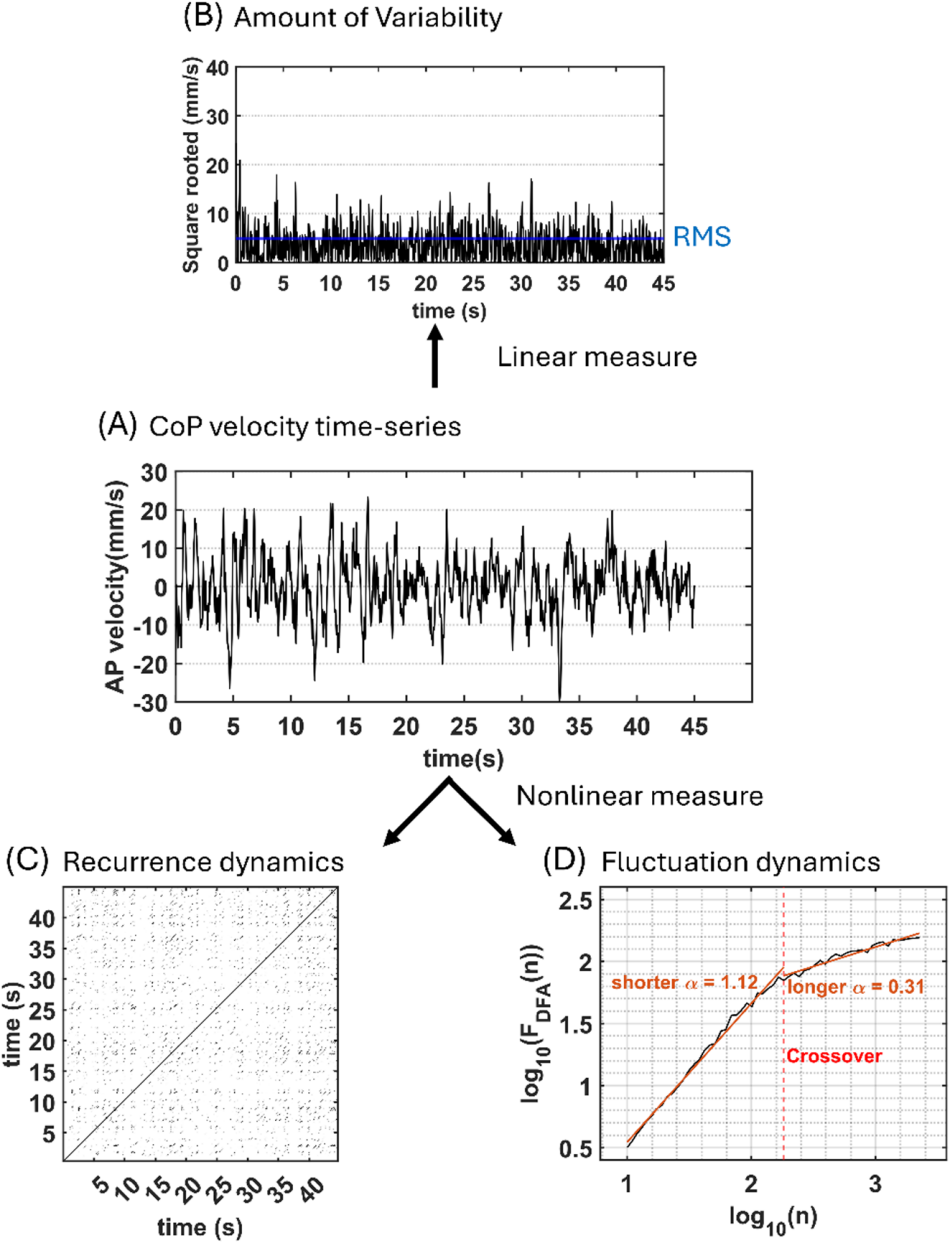
Overview of the data analysis: (**A**) Typical trial example of CoP velocity time-series data under the Visible Touch condition. (**B**) Squared time-series used for RMS calculation, with the blue line indicating the RMS value. (**C**) Recurrence plot generated from RQA. Dark points indicate recurrence points, where the trajectory in the reconstructed phase space returns to a previously visited state. White areas reflect non-recurrent regions. (**D**) Log-log plot used to calculate the scaling exponent α in DFA. The slope of the linear fit in the short-term region (left of the red line) represents the DFA-α for shorter time scales, while the slope in the long-term region (right of the red line) corresponds to the DFA-α for longer time scales.

In accordance with previous studies on interpersonal touch^5–7^, the RMS was used to quantify the variability of the CoP velocity time series, with smaller values indicating reduced variability and greater postural stability^30^. The RMS in the AP direction ($\:{RMS}_{AP}$) is defined as follows (Eq. 1) ^31^:

1 $$\:{RMS}_{AP}=\:\sqrt{\frac{1}{N}\sum\:_{t=1}^{N}{x\left(t\right)}^{2}}$$

where $\:x\:\left(t\right)$ represents the CoP velocity time series, with $\:t$ corresponding to each individual data point in the time series.$\:\:N$ is the total number of data points included in the analysis, which, in this study, was 4500 points. Figure 4A illustrates a representative example of the CoP velocity time series in the AP direction for one participant. In Fig. 4B, the time series represents the squared values of the original data used for RMS computation, with the solid blue line indicating the RMS values.

### Recurrence Quantification Analysis (RQA)

The RQA was employed to assess the recurrence dynamics of the CoP velocity time series. Specifically, the recurrence rate (%RECUR) and average diagonal line length (LMEAN) were used as indicators of the frequency and consistency of recurrence among similar patterns in the CoP velocity time series over time. RQA was implemented in three steps.

Step 1: Reconstruction of the Attractor.

In this step, the time delay parameter ($\:\tau\:$) and embedding dimension were determined. Two common approaches for selecting an appropriate $\:\tau\:$ involve identifying the first local minimum of the autocorrelation function (ACF) or the average mutual information (AMI) function^17^. However, owing to long-range correlations and non-stationarity, determining $\:\tau\:$ from the local minima of ACF or AMI in CoP time series data is challenging^17,32^. Our experimental data demonstrate this issue. Therefore, we adopted the approach proposed in a previous study^32^, in which τ is defined as the smallest delay at which the correlation between the CoP time series data $\:{X}_{i}$ and its time-delayed series $\:{X}_{i}+\tau\:$​ falls below a predefined threshold, indicating that the two series no longer exhibit strong linear dependence. Based on this method, $\:\tau\:$ was determined to be four samples (40 msec).

Selecting an appropriate embedding dimension is crucial because it must be sufficiently high to capture the interaction of a suitable number of dynamic variables. It is recommended to select a slightly higher embedding dimension, as insufficient dimensionality may fail to reconstruct the system’s dynamics correctly^16^. Previous studies on CoP time series have used embedding dimensions of 5 ^16^, 8 ^32^, and 10 ^17^. In this study, we selected an embedding dimension of 10, the upper end of the range reported in previous literature, to ensure that the complex interpersonal dynamics were fully captured without the risk of under-embedding.

Step 2: Construction of Recurrence Plots.

In this step, the radius parameter was determined, and a recurrence plot was generated. Recurrence is defined as the return of a trajectory in the reconstructed phase space to a sufficiently close position after a certain amount of time^33^. The radius serves as the threshold for defining recurrence. As RQA focuses on detecting local recurrences, an excessively large radius would classify several points as recurrent, which deviates from this principle^17^.

We calculated the %RECUR and LMEAN values across multiple radius values (0–100% of the mean CoP velocity time-series variability) while keeping the time delay and embedding dimension constant. The optimal radius was identified using a method that determined the range in which the relationship between radius, %RECUR, and LMEAN became linear on a logarithmic scale^32^. Additionally, previous studies indicated that the total recurrence rate should be maintained below 5% to ensure meaningful recurrence detection^17,33^. Based on these criteria, a radius of 45% of the mean CoP velocity time series variability was selected for this study. A recurrence plot was created based on these parameters, in which the dark points represent the recurrent points, as shown in Fig. 4C.

Step 3: Evaluation of Recurrence Points.

In this study, %RECUR and LMEAN were evaluated owing to their complementary roles in capturing distinct aspects of the temporal dynamics of postural control. %RECUR represents the proportion of recurrence points to the total number of possible points in the recurrence plot, expressed as a percentage. A high %RECUR value indicates that the system frequently revisits previous states, reflecting the presence of repetitive patterns over time. In contrast, a low %RECUR suggests a reduction in recurrence, which may reflect increased noise or random fluctuations within a time series^17^. However, a high or low recurrence rate alone does not necessarily indicate the regularity of the recurrence structure. The LMEAN, which is the average length of all continuous diagonal lines in the recurrence plot, was also assessed to address this limitation. The LMEAN reflects the mean duration for which similar states recur in sequence, thereby offering insights into the temporal consistency and stability of a system’s dynamics^22^. The combination of these two measures allows for a more comprehensive understanding of the temporal organization of postural sway. %RECUR captures the frequency with which the system revisits prior states, while LMEAN quantifies the consistency of those recurrences over time. Together, these indices provide a multifaceted representation of the regularity and stability underlying postural control.

### Detrended Fluctuation Analysis (DFA)

The DFA was employed to quantify the fluctuation dynamics of the CoP velocity time series. In this method, an integrated CoP velocity time series is first constructed by cumulatively summing the original data point by point. The series is then divided into segments of various lengths, local trends within each segment are removed, and the variance is calculated. The variance of the integrated CoP velocity time series increased as a function of the window length $\:n$, typically following a power-law relationship. The slope $\:\alpha\:$ of the double logarithmic plot of variance versus window length reflects the extent to which the time series maintains autocorrelation between temporally distant values, indicating the presence of long-range temporal dependencies or structure^19,34,35^. The DFA consists of three main steps.

Step 1: Calculation of Fluctuation Measure.

The first phase involves removing trends from the CoP time series data $\:x\left(t\right)$ to obtain the integrated time series denoted $\:X\left(t\right)$ (Eq. 2).

2 $$\:X\left(t\right)\:=\:\sum\:_{i=1}^{t}\left[x\left(i\right)-\stackrel{-}{x}\right]\:$$

where $\:\stackrel{-}{x}$ is the average of the entire time series, and $\:i$ represents the index of the time series. The fluctuation measure, $\:F\left(n\right)$, is then calculated from the recombined time series (Eq. 3).

3 $$\:F\left(n\right)=\:\sqrt{\frac{1}{N}\:\sum\:_{t=1}^{N}{\left[X\left(t\right)-\:{X}_{n}\left(t\right)\right]}^{2}}$$

where $$\:X\left(t\right)$$ is divided into nonoverlapping segments or windows of a specified length $$\:n$$. For each window, a regression line is fitted to eliminate any remaining trends, and the detrended data from each window are recombined into a new time series. This process is repeated for various window lengths constrained to the range $$\:10\:\le\:n\:\le\:\raisebox{1ex}{$N$}\!\left/\:\!\raisebox{-1ex}{$2$}\right.$$ ^36^. In this experiment, a maximum of 57 logarithmically spaced window sizes within the specified range were used and subsequently plotted on a log-log plot (Fig. 4D).

Step 2: Identification of Crossover.

Crossover refers to the phenomenon in which the slope of a double logarithmic plot changes at a specific timescale. The existence of a crossover suggests a transition in the temporal autocorrelation structure of the time series beyond a certain timescale, indicating that the correlation between temporally distant points in the CoP velocity time series shifts from persistent to anti-persistent patterns^19^.

In this study, the optimal crossover point was determined as the point at which the sum of squared residuals for the short- and long-term regression lines was minimized. This optimal point was established by averaging the four conditions. As changing the crossover value individually for each condition could introduce distortions into the data, use of a unified crossover value across all conditions was considered appropriate. Ultimately, the crossover value was determined to be log₁₀(2.26). Time scales below the crossover point were classified as DFA shorter $\:\alpha\:$, while those above were classified as DFA longer $\:\alpha\:$ (Fig. 4D).

Step 3: Calculation of the Scaling Exponent $\:\alpha\:$.

The final step involves calculating the slope obtained from the log-log plot, denoted as $\:\alpha\:$ (Eq. 4).

4 $$\:\mathrm{F}\left(\mathrm{n}\right)\:\propto\:{\mathrm{n}}^{\alpha\:}$$

The scaling exponent $\:\alpha\:$, obtained through DFA, serves as an indicator of both the persistence of temporal correlations and the stationarity characteristics of a time series across multiple time scales. An $\:\alpha\:$ value equal to 0.5 indicates that the signal behaves as uncorrelated white noise, reflecting the absence of long-range temporal correlations. When $\:\alpha\:$ is greater than 0.5, the time series exhibits persistent dynamics. This finding implies that, if the signal increases at a given point in time, it is likely to continue to increase. Similarly, if the signal decreases, the decrease is likely to continue. In contrast, when $\:\alpha\:$ is less than 0.5, the signal demonstrates anti-persistent dynamics, in which increases are more likely to be followed by decreases, and vice versa.

In addition, the value of $\:\alpha\:$ provides insight into the stationarity of the signal^19^. Time series with $\:\alpha\:$ values below 1 are considered stationary, indicating statistical properties that remain constant over time. Values of $\:\alpha\:$ exceeding 1 signify non-stationary dynamics, in which fluctuations tend to drift, meaning their statistical characteristics continuously change and progressively deviate from equilibrium. An $\:\alpha\:$ value close to 1 represents the boundary between stationary and non-stationary behavior and is often referred to as pink noise, which is characterized by long-range temporal correlations without sustained divergence.

### Statistical analysis

A two-way repeated-measures ANOVA with two within-subject factors, partner visibility (Visible vs. Invisible) and interpersonal tactile input (Touch vs. No Touch), was performed on the RMS, %RECUR, LMEAN, shorter α, and longer α. For each participant and condition, the mean value of each measure across the five trials was used as a representative value. When significant main effects or interactions were found, *post hoc* simple main effects tests were performed to examine the individual effects of each factor. In cases where the sphericity assumption was violated, the Greenhouse-Geisser correction was applied (Mendoza’s test, *p* = 0.0005). Effect sizes were quantified as partial eta-squared (ηp²), calculated as the ratio of the sum of squares for each effect to the total sum of squares. Statistical significance was set at *p* < 0.05.

## Data Availability

The datasets used and/or analyzed during the current study are not publicly available due to participant privacy concerns but are available from the corresponding author on reasonable request.
